# Glycolytic ATP Fuels the Plasma Membrane Calcium Pump Critical for Pancreatic Cancer Cell Survival*

**DOI:** 10.1074/jbc.M113.502948

**Published:** 2013-10-24

**Authors:** Andrew D. James, Anthony Chan, Oihane Erice, Ajith K. Siriwardena, Jason I. E. Bruce

**Affiliations:** From the ‡ Faculty of Life Sciences, The University of Manchester, Michael Smith Building, Oxford Road, Manchester, M13 9PT, United Kingdom and; the § Hepatobiliary Surgery Unit, Manchester Royal Infirmary, Manchester, M13 9NT, United Kingdom

**Keywords:** ATP, Calcium ATPase, Calcium Signaling, Cell Death, Glycolysis, Metabolism, Pancreatic Cancer, PMCA, Warburg, Calcium Overload

## Abstract

Pancreatic cancer is an aggressive cancer with poor prognosis and limited treatment options. Cancer cells rapidly proliferate and are resistant to cell death due, in part, to a shift from mitochondrial metabolism to glycolysis. We hypothesized that this shift is important in regulating cytosolic Ca^2+^ ([Ca^2+^]*_i_*), as the ATP-dependent plasma membrane Ca^2+^ ATPase (PMCA) is critical for maintaining low [Ca^2+^]*_i_* and thus cell survival. The present study aimed to determine the relative contribution of mitochondrial *versus* glycolytic ATP in fuelling the PMCA in human pancreatic cancer cells. We report that glycolytic inhibition induced profound ATP depletion, PMCA inhibition, [Ca^2+^]*_i_* overload, and cell death in PANC1 and MIA PaCa-2 cells. Conversely, inhibition of mitochondrial metabolism had no effect, suggesting that glycolytic ATP is critical for [Ca^2+^]*_i_* homeostasis and thus survival. Targeting the glycolytic regulation of the PMCA may, therefore, be an effective strategy for selectively killing pancreatic cancer while sparing healthy cells.

## Teaser

**Background:** Pancreatic cancer cells exhibit up-regulated glycolysis.

**Results:** Inhibition of glycolysis, but not mitochondrial metabolism, induced ATP depletion, plasma membrane calcium pump (PMCA) inhibition, Ca^2+^ overload, and cell death.

**Conclusion:** Glycolytic ATP fuels the PMCA in pancreatic cancer.

**Significance:** Glycolytic regulation of the PMCA may represent a novel chemotherapeutic target for pancreatic cancer.

## Introduction

Pancreatic ductal adenocarcinoma (PDAC)^3^ is an aggressive form of cancer that originates in the glandular ductal tissue of the exocrine pancreas. Treatment options are severely limited, with surgical removal of the tumor being the most common course of action. PDAC has a severe mortality rate, which as a proportion of incidence is almost 100% (1); pancreatic cancer is the forth and fifth most common cause of cancer-related death in men and women, respectively. PDAC often progresses to metastasis in the absence of obvious clinical symptoms (2), resulting in late diagnosis and a 5-year survival rate of 1% (3).

A hallmark of cancer cells, including PDAC, is a shift from predominantly mitochondrial metabolism toward glycolysis, even when oxygen is abundant (the “Warburg effect”; Refs. 4 and 5). Such a metabolic shift seems counterintuitive, as the textbook view is that glycolytic ATP synthesis is an energetically unfavorable method of meeting the presumably high ATP demand of rapidly proliferating cancer cells. However, a shift toward glycolysis appears to confer a number of survival advantages for tumor cells (6). These include resistance to hypoxia, which is prevalent in PDAC tumors (7), and an increased availability of glycolytic intermediates for use in the anabolic pathways that drive cell proliferation (8).

It is, therefore, unlikely that cancer cells exhibit the Warburg phenotype primarily for bioenergetic purposes despite exhibiting an increased reliance on glycolytic ATP (9). However, the homeostatic maintenance of a low resting cytosolic calcium (Ca^2+^) concentration ([Ca^2+^]*_i_*, ∼100 nm) is an ATP-dependent process that is critical for cell survival. Of particular importance is the ubiquitously expressed plasma membrane Ca^2+^ ATPase (PMCA), an ATP-dependent pump with a high affinity for Ca^2+^ that extrudes cytosolic Ca^2+^ (10). The PMCA has been suggested as the major efflux pathway in non-excitable cells (such as epithelial cells) where the Na^+^/Ca^2+^ exchanger (NCX) is either lacking or expressed with low abundance (11). In these cells impaired PMCA function quickly results in [Ca^2+^]*_i_* overload and cell death, indicating that PMCA function is critical for cell survival. Under physiological conditions when ATP is abundant, the source of ATP to fuel the PMCA is not likely to be important provided that the cytosolic ATP is maintained above a critical threshold. The classical view is that the bulk of ATP comes from the mitochondria, and evidence suggests that inhibition of mitochondrial metabolism in non-cancerous cells impairs Ca^2+^ homeostasis and leads to cell death (12–14). However, in cancer cells where there is a shift toward glycolytic metabolism, this relationship may be very different. Importantly, the PMCA has been reported to have its own localized glycolytic ATP supply (15, 16). It could, therefore, be hypothesized that glycolytic ATP is critical for fuelling the PMCA and confers a survival advantage to cancer cells.

The present study shows that in human PDAC cell lines (PANC1 and MIA PaCa-2), inhibition of glycolysis induced severe ATP depletion, cytosolic Ca^2+^ overload, inhibition of PMCA activity, and cell death. In contrast, inhibition of mitochondrial metabolism had almost no effect on [Ca^2+^]*_i_* handling, ATP depletion, or cell death. Glycolytic regulation of the PMCA may, therefore, be a critical pro-survival mechanism in PDAC and thus may represent a previously untapped therapeutic avenue for selectively killing PDAC cells while sparing normal cells.

## EXPERIMENTAL PROCEDURES

### 

#### 

##### Cell Culture

MIA PaCa-2 and PANC1 cells (ATCC) were grown in DMEM (D6429, Sigma, supplemented with 10% FBS, 100 units/ml penicillin, and 100 μg/ml streptomycin) in a humidified atmosphere of air/CO_2_ (95%:5%) at 37 °C. Cells were used up to passage 30 and then discarded.

##### Fura-2 Fluorescence Ca^2+^ Imaging

Cells were seeded onto glass coverslips in a 6-well culture plate and grown to >30% confluency. To load cells with fura-2 dye, seeded coverslips were rinsed with HEPES-buffered physiological saline solution (HEPES-PSS; 138 mm NaCl, 4.7 mm KCl, 1.28 mm CaCl_2_, 0.56 mm MgCl_2_, 5.5 mm glucose, 10 mm HEPES, pH 7.4). Rinse buffer was replaced with 4 μm fura-2 AM in 1 ml HEPES-PSS and incubated for 40 min at room temperature. Cells were then rinsed with HEPES-PSS followed by a further 20 min in dye-free HEPES-PSS to allow uncleaved dye to re-equilibrate. Fura-2-loaded cells were mounted onto imaging systems, and [Ca^2+^]*_i_* was measured as previously described (12, 17). Experiments were performed using a Nikon Diaphot fitted with a ×40 oil immersion objective (numerical aperture 1.3) and an Orca CCD camera (Hamamatsu), whereas the PANC1 [Ca^2+^]*_i_* clearance assays were performed using a Nikon TE2000 microscope fitted with a ×40 oil immersion objective (numerical aperture 1.3) and a CoolSNAP HQ interline progressive-scan CCD camera (Roper Scientific Photometrics, Tucson, AZ). Both systems used a monochromator illumination system (Cairn Research, Kent, UK) and were controlled by MetaFluor image acquisition and analysis software (Molecular Devices, Downingtown, PA). Cells were continually perfused with HEPES-PSS using a gravity-fed perfusion system (Harvard apparatus) and were excited at 340 and 380 nm (50-ms exposure). Emitted light was separated from excitation using a 400-nm dichroic with 505LP filter. Background-subtracted images of a field of view of cells were acquired every 5 s for both excitation wavelengths (340 and 380 nm). For all experiments, [Ca^2+^]*_i_* was measured as fura-2 340/380 nm fluorescence ratio. [Ca^2+^]*_i_* clearance was measured using an *in situ* [Ca^2+^]*_i_* clearance assay as previously described (18). Unless stated, 0 Ca^2+^ HEPES-PSS contained 1 mm EGTA. Experiments (between 5 and 32 cells) were performed at room temperature.

##### Preparation of Test Reagents

Na^+^-free HEPES-PSS was prepared by replacing Na^+^ with equimolar *N*-methyl d-glucamine (Na^+^-free/NMDG). Stocks of La^3+^, 3-bromopyruvate (BrPy), sodium iodoacetate (IAA), and ATP (Mg^2+^ salt) were prepared in MilliQ water. Stocks of oligomycin (OM), carbonyl cyanide *m*-chlorophenyl hydrazine (CCCP), and cyclopiazonic acid (CPA) were prepared in DMSO. Fura-2 AM (Invitrogen, TEFLabs) was prepared in 50:50 DMSO (Sigma), and 0.1% Pluronic® F-127 (Molecular Probes, Invitrogen). Working solutions in HEPES-PSS were prepared from frozen stocks immediately before an experiment. Reagents were prepared in media for the ATP and cell death experiments.

##### Cell Death Assays

PANC1 cells were seeded into black-walled, clear-bottom 96-well plates at 70% confluency and allowed to adhere overnight. Cells were then incubated in culture conditions with blank media or ascending concentrations of CCCP (1–10 μm), BrPy (100–1000 μm), or a combination (3 and 500 μm, respectively) for 0.5–6 h. Cells were then stained with propidium iodide (PI, cell death, 2 μg/ml, Fluka) and Hoechst 33342 (cell count, 20 μg/ml; Invitrogen). PI-positive cell count was normalized to Hoechst 33342-positive cell count (%). Assays were run in duplicate on a Thermo Fisher Scientific Cellomics® ArrayScan® VTI HCS Reader fitted with Hamamatsu ORCAR-ER camera and both ×10 and ×20 objectives. Imaging was performed by Imagen Biotech Ltd. Image analysis was assessed using the Compartmental Analysis Bioapplication (Thermo Fisher Scientific) whereby a threshold gate was set (∼500 levels of gray) along an intensity histogram plot to distinguish positive PI fluorescent cells (∼5000 levels of gray) from background noise (∼10 levels of gray).

##### ATP Measurements

Cultured MIA PaCa-2 and PANC1 cells were seeded into white-walled, clear-bottom 96-well plates (1 × 10^5^ cells/ml) and allowed to adhere overnight. Cells were then treated with varying concentrations of metabolic inhibitors and incubated for 15 min in culture conditions. Each experiment included untreated cells (control), which represented total ATP, and a positive control with cells treated with an ATP depletion mixture (4 μm CCCP, 10 μm OM, 500 μm BrPy, 2 mm IAA) to achieve maximal ATP depletion. After treatment, cells were lysed, and ATP was determined using the luciferase-based ViaLight® Plus kit (Lonza, Rockland, ME). Luminescence was measured using a Synergy HT multiwell reader (BioTEK). Each experiment was run in duplicate; the luminescence counts of each duplicate pair were averaged. To correct for background luminescence under conditions of maximal ATP depletion, the averaged luminescence count from the ATP depletion mixture duplicates was subtracted from all other values. These values were normalized to the corresponding control (%).

##### Calibration of Resting [Ca^2+^]*_i_*

[Ca^2+^]*_i_* calibrations were performed by first applying 10 μm ionomycin in the absence of external Ca^2+^ to naïve fura-2 loaded PANC1 ( *n* = 30 cells), and MIA PaCa-2 cells (*n* = 25 cells). Once [Ca^2+^]*_i_* reached a minimum (*R*_min_), cells were perfused with 20 mm Ca^2+^ to induce a maximum increase in [Ca^2+^]*_i_* (*R*_max_). Fura-2 ratios were then calibrated to determine [Ca^2+^]*_i_* as previously described (19). Fura-2 ratios were plotted against calibrated log[Ca^2+^]*_i_*, with all cells from each cell line treated as a single data series. A single sigmoidal curve was then fitted representative of calibrated [Ca^2+^]*_i_* in an average cell. The equation derived from this curve was used to estimate [Ca^2+^]*_i_* and was extrapolated for each cell line. 100 μm ATP was used to test cell viability, with viable cells eliciting a [Ca^2+^]*_i_* spike.

##### Measurement of [Ca^2+^]*_i_* Clearance

Repeated measurements of [Ca^2+^]*_i_* clearance rate were performed in parallel on cells from the same passage in the presence or absence of test reagents during the second [Ca^2+^]*_i_* clearance phase. The linear clearance rate over 60 s for the first influx-clearance phase was determined in fura-2 ratio units/second. This was repeated for the second influx-clearance phase (measured from the same standardized fura-2 value), and the second rate was normalized to the first. Values were averaged for all cells in an experiment, and the resulting experimental means for each condition were averaged to give the presented group means ± S.E.

##### Data Analysis

Cell death was statistically assessed using a two-way analysis of variance with a post hoc Bonferroni correction. Changes in resting [Ca^2+^]*_i_* were quantified by measuring the area under the curve (AUC) and the maximum increase in [Ca^2+^]*_i_* over 30 min and compared using an unpaired Student's *t* test. Cell viability was quantified as the percentage of cells responding to 100 μm ATP and compared using a Mann-Whitney *U* test. Changes in [Ca^2+^]*_i_* clearance rate were compared using a Mann-Whitney *U* test. ATP depletion was assessed using a one-sample *t* test against the hypothetical control value of 100%. Presented data are the means ± S.E. of the indicated number (*n*) of independent experiments.

## RESULTS

### 

#### 

##### Inhibition of Glycolysis but Not Mitochondrial Metabolism Induces Cell Death in Human PANC1 Cells

To determine whether PANC1 cells functionally exhibit the Warburg effect with respect to cell viability, the effects of both a mitochondrial and a glycolytic inhibitor on cell death were measured using PI fluorescence. PANC1 cells were treated for 0.5–6 h with either BrPy, an inhibitor of the glycolytic enzyme hexokinase (20), or CCCP, a protonophore that collapses the mitochondrial membrane potential (18).

**FIGURE 1.**
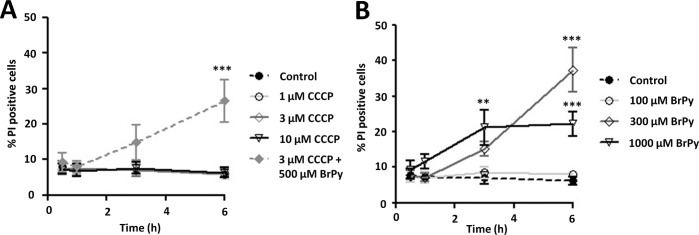
**Inhibition of glycolysis, but not mitochondrial metabolism, induces cell death in pancreatic cancer cells.** PANC1 cells were treated with either the mitochondrial inhibitor, CCCP (1–10 μm, *A*), the glycolytic inhibitor, BrPy (100–1000 μm, *B*), or a combination of both (*A*, *filled diamond*) for varying times (0.5–6 h). Cell death was determined using PI fluorescence on a Cellomics® ArrayScan® VTI HCS Reader (Imagen Biotech). PI-positive cells (dead) were normalized to Hoechst-positive cells (cell count) to determine % cell death (*n* = 5). **, *p* < 0.01; ***, *p* < 0.001 compared with control (two-way analysis of variance, post hoc Bonferroni correction).

Inhibition of mitochondrial metabolism with all three concentrations of CCCP (1, 3, and 10 μm) had no effect on cell death at any time point compared with corresponding control experiments (Fig. 1*A*, *n* = 5). In contrast, inhibition of glycolysis with BrPy resulted in a time-dependent increase in cell death at higher concentrations of BrPy. After 6 h of treatment, 300 μm BrPy caused significant cell death, with 37 ± 6% of cells positive for PI (*n* = 5) compared with 6 ± 1% for the corresponding control cells (Fig. 1*B*, *p* < 0.001, *n* = 5). Similar effects were observed at higher concentrations of BrPy and longer treatment periods. The combination of 3 μm CCCP and 500 μm BrPy caused a similar cell death to BrPy alone over a similar time frame, with 27 ± 6% of cells staining for PI (Fig. 1*A*, *p* < 0.001, *n* = 5); no difference in cell death was observed between 6 h of treatment with 300 μm BrPy alone or a combination of 3 μm CCCP and 500 μm BrPy. These results strongly suggest that PANC1 cells are sensitive to inhibition of glycolysis by BrPy yet are unaffected by inhibition of mitochondrial metabolism by CCCP.

##### Inhibitors of Glycolytic but Not Mitochondrial Metabolism Induce Cytosolic Ca^2+^ Overload in Human PDAC Cells

We next tested the effects of glycolytic and mitochondrial inhibitors on resting [Ca^2+^]*_i_* in the human PDAC cell lines PANC1 and MIA PaCa-2 using fura-2 fluorescence imaging. The PANC1 and MIA PaCa-2 cell lines were selected because these cell lines express oncogenic KRAS mutations at codon 12 (21). This mutation is a key hallmark of clinical PDAC (22) and has been shown to both initiate and drive PDAC in transgenic mouse models (23). Inhibition of glycolytic metabolism was achieved using IAA (2 mm), which inhibits glyceraldehyde-3-phosphate dehydrogenase (24) or BrPy (500 μm). Conversely, inhibition of mitochondrial ATP production was achieved using either OM (10 μm), an inhibitor of the mitochondrial F_1_/F_0_-ATP synthase (25), or CCCP (4 μm). Cells were continuously perfused with HEPES-PSS, and metabolic inhibitors were applied for 30 min. After 30 min of treatment cells were treated with 100 μm ATP to activate purinergic receptor-induced [Ca^2+^]*_i_* responses, thereby testing for reversibility of responses and thus viability.

**FIGURE 2.**
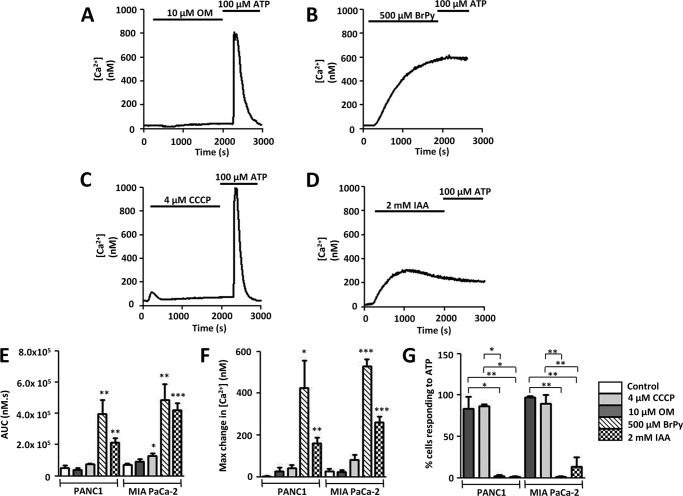
**Glycolytic inhibitors but not mitochondrial inhibitors induce an irreversible cytosolic Ca^2+^ overload in pancreatic cancer cells.** Using fura-2 fluorescence imaging, [Ca^2+^]*_i_* concentration was measured in PANC1 or MIA PaCa cells. *A–D*, representative traces show the effect of various metabolic inhibitors on [Ca^2+^]*_i_* in PANC1 cells. Cells were treated for 30 min with either mitochondrial inhibitors (10 μm OM (*A*); 4 μm CCCP (*C*)) or glycolytic inhibitors (500 μm BrPy (*B*); 2 mm IAA (*D*)) followed by stimulation with the purinergic agonist, ATP (100 μm) to test for cell viability. Similar qualitative results were obtained for MIA PaCa-2 cells. Responses were quantified by measuring the AUC (*E*) for the 30-min treatment with drug and maximum change in [Ca^2+^]*_i_* (*F*). Recovery from metabolic inhibitor treatment was assessed by measuring the % cells that subsequently responded to ATP (*G*). *, *p* < 0.05; **, *p* < 0.01; ***, *p* < 0.001 (*E* and *F*, unpaired Student's *t* test; *G*, Mann-Whitney *U* test) compared with control.

Treatment with BrPy (500 μm) for 30 min induced an irreversible [Ca^2+^]*_i_* overload, with an average maximum increase in [Ca^2+^]*_i_* of 531 ± 32 nm in MIA PaCa-2 cells (*n* = 5) and 425 ± 134 nm in PANC1 cells (*n* = 5, Fig. 2, *B* and *F*). Similarly, treatment with 2 mm IAA induced an average maximum increase in [Ca^2+^]*_i_* of 261 ± 29 nm in MIA PaCa-2 cells (*n* = 7) and 160 ± 26 nm in PANC1 cells (*n* = 6, Fig. 2, *D* and *F*). These responses were significant compared with corresponding time-matched control cells for both MIA PaCa-2 (26 ± 12 nm, *n* = 5, *p* < 0.001 for both BrPy and IAA, unpaired Student's *t* test) and PANC1 cells (3 ± 2 nm, *n* = 4, *p* < 0.05 for BrPy, *p* < 0.01 for IAA, unpaired Student's *t* test).

Similarly, BrPy (500 μm) increased the AUC to 485 ± 104 μm·s in MIA PaCa-2 cells and 399 ± 86 μm·s in PANC1 cells, whereas IAA (2 mm) elevated the AUC to 421 ± 44 μm·s in MIA PaCa-2 cells and 213 ± 28 μm·s in PANC1 cells (Fig. 2*E*). These responses were significantly elevated compared with control MIA PaCa-2 (71 ± 9 μm·s; BrPy, *p* < 0.01; IAA, *p* < 0.001, unpaired Student's *t* test) and PANC1 cells (52 ± 14 μm·s, *p* < 0.01, unpaired Student's *t* test). AUC represents a measure of not only the magnitude of [Ca^2+^]*_i_* increase but also the recovery of response.

In contrast, OM (10 μm, Fig. 2*A*) and CCCP (4 μm, Fig. 2*C*) had no effect on the maximum increase in [Ca^2+^]*_i_* observed in either MIA PaCa-2 cells (Fig. 2*F*, *n* = 7 and *n* = 5, respectively) or PANC1 cells (Fig. 2*F*, *n* = 4 and *n* = 4, respectively). Interestingly, CCCP elicited a small but significant increase in AUC to 128 ± 16 μm·s (*n* = 5) in MIA PaCa-2 cells (*n* = 5, *p* < 0.05, unpaired Student's *t* test) but not in PANC1 cells (*n* = 4, Fig. 2*E*). Despite this, OM had no effect on AUC in either MIA PaCa-2 or PANC1 cells (*n* = 7 and n-4, respectively, Fig. 2*E*).

In addition to their effects on [Ca^2+^]*_i_*, BrPy (500 μm) and IAA (2 mm) abolished the ability of both cell lines to elicit Ca^2+^ responses to 100 μm ATP (Fig. 2, *B*, *D*, and *G*). In healthy cells, ATP evoked robust spike-like increases in [Ca^2+^]*_i_*, indicating that these cells were viable. In contrast to the glycolytic inhibitors, ATP-evoked Ca^2+^ responses were observed in the majority of cells treated with the mitochondrial inhibitors OM (10 μm, Fig. 2*A*) or CCCP (4 μm, Fig. 2*C*). Together these results suggest that glycolysis, but not mitochondrial metabolism, is critically important for maintaining a low resting [Ca^2+^]*_i_* in human PDAC cells.

##### Validation That in Situ [Ca^2+^]*_i_* Clearance Assay Represents PMCA Activity in PDAC

We previously developed an *in situ* [Ca^2+^]*_i_* clearance assay in pancreatic acinar cells in which PMCA activity is pharmacologically and functionally isolated (12, 18). Briefly, sarcoplasmic/endoplasmic reticulum Ca^2+^ ATPase (SERCA) activity was blocked by continuous application of CPA in the absence of extracellular Ca^2+^ and the presence of 1 mm EGTA, leading to endoplasmic reticulum Ca^2+^ store depletion. Rapid store-operated Ca^2+^ entry was then induced upon perfusion with 20 mm Ca^2+^. The subsequent removal of extracellular Ca^2+^ resulted in clearance of [Ca^2+^]*_i_*, presumably via the PMCA. This influx-clearance phase was repeated, and test reagents or maneuvers were applied during the second influx-clearance phase. Thus, the resulting paired experimental design controlled for cell-to-cell and temporal variability.

**FIGURE 3.**
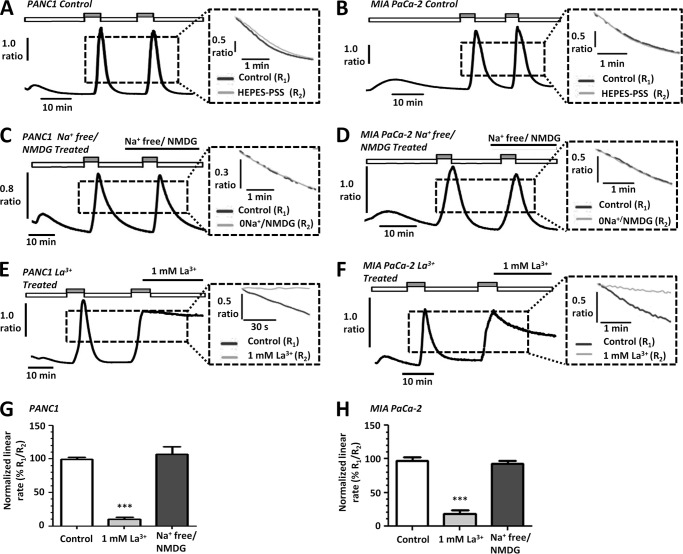
**PMCA is the main mechanism of [Ca^2+^]*_i_* efflux in human PDAC cell lines.** *A–F*, representative traces show the *in situ* [Ca^2+^]*_i_* clearance assay (PMCA activity) for control PANC1 (*A*) and MIA PaCa-2 (*B*) cells, Na^+^-free/NMDG-treated PANC1 (*C*) and MIA PaCa-2 (*D*) cells, and La^3+^ treated PANC1 (*E*) and MIA PaCa-2 cells (*F*). Cells were treated with CPA (30 μm) in zero external Ca^2+^ with 1 mm EGTA (*white box*) or 20 mm Ca^2+^ (*gray box*) to induce store-operated Ca^2+^ influx. Subsequent removal of external Ca^2+^ resulted in [Ca^2+^]*_i_* clearance. This influx-clearance phase was repeated under conditions where extracellular Na^+^ was replaced with equimolar Na^+^-free/NMDG or in the presence of 1 mm La^3+^. La^3+^ was prepared in HEPES-PSS devoid of EGTA to prevent chelating of the La^3+^. The *inset* of each trace shows expanded time courses comparing the second (*gray trace*) with the first clearance phase (*black trace*) in the presence of each treatment. The linear clearance rate over 60 s (in the presence of each treatment) was normalized to the initial clearance rate in each cell (% relative clearance). Normalized linear rate (± S.E.) is presented for PANC1 (*G*) and MIA PaCa-2 (*H*) cells. ***, *p* < 0.001 (Mann-Whitney *U* test) compared with time-matched control experiments (*white bar*).

[Ca^2+^]*_i_* clearance can be quantified either by fitting the falling phase to a single exponential decay or by measuring the initial linear clearance rate from a standardized [Ca^2+^]*_i_* value. However, fitting to an exponential decay is only valid if the [Ca^2+^]*_i_* clears to approximately the same base-line [Ca^2+^]*_i_* (asymptote) and is not accurate if clearance approaches a slow linear phase. This was almost always the case when clearance was severely inhibited. We, therefore, normalized the linear clearance rate over 60 s during the second clearance phase to that of the first (%), both measured from the same [Ca^2+^]*_i_* value. Before assessing the effects of metabolic inhibitors on [Ca^2+^]*_i_* clearance during our *in situ* [Ca^2+^]*_i_* clearance assay, control experiments revealed that clearance rate was maintained between sequential influx-clearance phases in untreated PANC1 (100 ± 3%, *n* = 18, Fig. 3, *A* and *G*) and MIA PaCa-2 cells (98 ± 5%, *n* = 17, Fig. 3, *B* and *H*).

Although the NCX is not thought to contribute to [Ca^2+^]*_i_* clearance in pancreatic acinar cells (26–28), evidence suggests it may play a role in rat pancreatic ductal cells (29, 30). Furthermore, NCX is reported to be expressed in human PDAC cells (30–32). To determine whether NCX contributed to Ca^2+^ clearance in PANC1 and MIA PaCa-2 cells under the conditions of our *in situ* [Ca^2+^]*_i_* clearance assay, we replaced extracellular Na^+^ with equimolar *N*-methyl d-glucamine (Na^+^-free/NMDG). NMDG is not transported by NCX yet maintains the osmotic balance, thereby removing the Na^+^ gradient required for NCX-mediated Ca^2+^ efflux.

Na^+^-free/NMDG had no effect on [Ca^2+^]*_i_* clearance in PANC1 (Fig. 3, *C* and *G*, *n* = 7) or MIA PaCa-2 cells (Fig. 3, *D* and *H*, *n* = 4) compared with control cells (PANC1, Fig. 3*A*, *n* = 18; MIA PaCa-2, Fig. 3*B*, *n* = 17). These data indicate that NCX does not contribute to [Ca^2+^]*_i_* clearance in these cell lines under the current experimental conditions.

Furthermore, to test whether the PMCA is responsible for [Ca^2+^]*_i_* clearance in PANC1 and MIA PaCa-2 cells during the *in situ* [Ca^2+^]*_i_* clearance assay, 1 mm La^3+^ was applied during the second clearance phase. La^3+^ inhibits Ca^2+^ efflux (PMCA) at millimolar concentrations (33). However, because La^3+^ also inhibits store-operated Ca^2+^ entry at micromolar concentrations (34), La^3+^ was applied at the peak of the Ca^2+^ influx, first for 1 min in the presence of 20 mm Ca^2+^ before removal of external Ca^2+^ under the continued application of La^3+^. These experiments were performed using HEPES-PSS devoid of EGTA as this also chelates La^3+^ (35). 1 mm La^3+^ dramatically inhibited the linear clearance rate to 10 ± 3% in PANC1 cells (Fig. 3, *E* and *G*, *n* = 5) and 18 ± 5% in MIA PaCa-2 cells (Fig. 3, *F* and *H*, *n* = 7) compared with 100 ± 3% (*n* = 18) and 98 ± 5% (*n* = 17) in corresponding time-matched control cells, respectively (PANC1, Fig. 3, *A* and *G*; MIA PaCa-2, Fig. 3, *B* and *H*; *p* < 0.001, Mann-Whitney *U* test).

Collectively these data confirm that the PMCA is the major Ca^2+^ clearance pathway and that NCX plays no role in [Ca^2+^]*_i_* clearance in these cells under these conditions. Any inhibition of [Ca^2+^]*_i_* clearance observed using this experimental design can, therefore, be interpreted as an effect on PMCA activity, consistent with our previous studies (12, 18).

##### Inhibitors of glycolytic but Not Mitochondrial Metabolism Inhibit PMCA Activity in Human PDAC Cells

**FIGURE 4.**
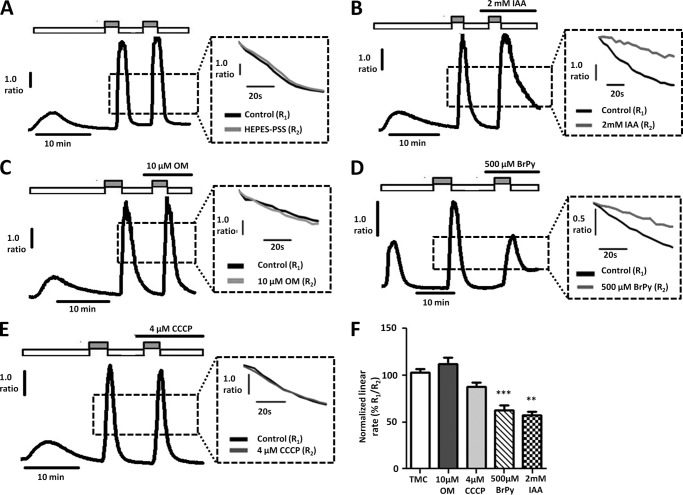
**Glycolytic inhibitors, but not mitochondrial inhibitors, inhibit PMCA activity in PANC1 cells.** *A–E*, representative traces showing the *in situ* [Ca^2+^]*_i_* clearance assay (PMCA activity) in fura-2-loaded PANC1 cells. CPA (30 μm) was applied in zero external Ca^2+^ with 1 mm EGTA (*white box*) or 20 mm Ca^2+^ (*gray box*) to induce store-operated Ca^2+^ influx. Subsequent removal of external Ca^2+^ resulted in [Ca^2+^]*_i_* clearance. This influx-clearance phase was repeated using a paired experimental design, and metabolic inhibitors were applied during this second influx-clearance phase. Each *inset trace* shows expanded time courses comparing the second (*gray trace*) with the first clearance phase (*black trace*) in the presence of each metabolic inhibitor. *A*, time-matched control (TMC); *B*, 2 mm IAA; *C*, 10 μm OM; *D*, 500 μm 3-BrPy; *E*, 4 μm CCCP. Linear clearance rate over 60 s during the second clearance phase was normalized to that of the first (% relative clearance). *F*, normalized linear rate (± S.E.). **, *p* < 0.01; ***, *p* < 0.001 (Mann-Whitney *U* test), compared with TMC.

After establishing that inhibition of glycolysis results in [Ca^2+^]*_i_* overload, we aimed to determine whether this was due at least in part to inhibition of the PMCA. To test this, we applied the glycolytic inhibitors (IAA (2 mm) and BrPy (500 μm)) or mitochondrial inhibitors (OM (10 μm) and CCCP (4 μm)) during the second Ca^2+^ clearance phase of our *in situ* [Ca^2+^]*_i_* clearance assay. In PANC1 cells, both IAA and BrPy markedly decreased PMCA activity to 57 ± 4% (Fig. 4, *B* and *F*, *n* = 5) and 63 ± 5% (Fig. 4, *D* and *F*, *n* = 8), respectively, compared with time-matched control cells (103 ± 4%, Fig. 4, *A* and *F*, *n* = 11, *p* < 0.005, Mann-Whitney *U* test). In contrast, the mitochondrial inhibitors CCCP and OM had no effect (CCCP, *n* = 8, Fig. 4, *E* and *F*; OM, *n* = 5, Fig. 4, *C* and *F*).

**FIGURE 5.**
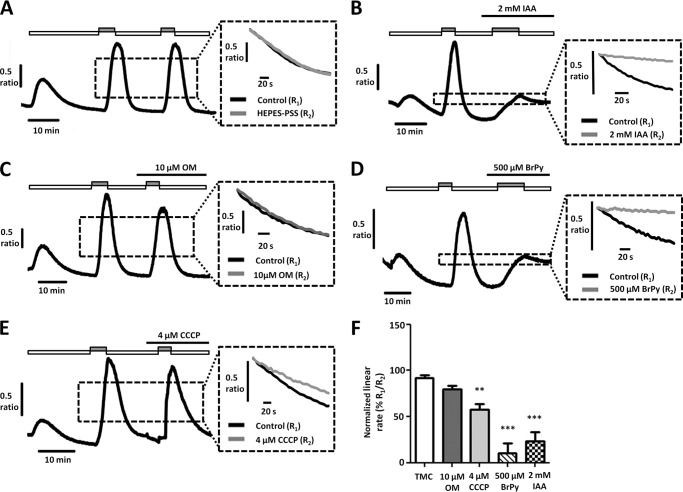
**Glycolytic inhibitors, but not mitochondrial inhibitors, inhibit PMCA activity and store-operated Ca^2+^ entry in MIA PaCa-2 cells.** *A–E*, representative traces show the *in situ* [Ca^2+^]*_i_* clearance assay (PMCA activity) in fura-2-loaded MIA PaCa-2 cells. CPA (30 μm) was applied in zero external Ca^2+^ with 1 mm EGTA (*white box*) or 20 mm Ca^2+^ (*gray box*) to induce store-operated Ca^2+^ influx. Subsequent removal of external Ca^2+^ resulted in [Ca^2+^]*_i_* clearance. This influx-clearance phase was repeated using a paired experimental design, and metabolic inhibitors were applied during this second influx-clearance phase. The *inset* of each trace shows expanded time courses comparing the second (*gray trace*) with the first clearance phase (*black trace*) in the presence of each metabolic inhibitor. *A*, TMC; *B*, 2 mm IAA; *C*, 10 μm OM; D, 500 μm BrPy; *E*, 4 μm CCCP. Linear clearance rate over 60 s during the second clearance phase was normalized to that of the first (% relative clearance). *F*, normalized linear rate (± S.E.). **, *p* < 0.01; ***, *p* < 0.001 (Mann-Whitney *U* test), compared with TMC.

Similarly, in MIA PaCa-2 cells, IAA (2 mm) and BrPy (500 μm) also inhibited PMCA activity. We first attempted to repeat the [Ca^2+^]*_i_* clearance experiments in MIA PaCa-2 cells using an identical protocol to that used for PANC1 cells. BrPy (500 μm, Fig. 5*D*) and IAA (2 mm, Fig. 5*B*) both significantly decreased PMCA activity to 10 ± 10% (*n* = 4) and 23 ± 10% (*n* = 5, Fig. 5*F*), respectively, compared with the corresponding time-matched control cells (92 ± 3%, *n* = 6, *p* < 0.001 for both BrPy and IAA, Mann-Whitney *U* test). Interestingly, CCCP decreased PMCA activity to 58 ± 6% (Fig. 5, *E* and *F*, *p* < 0.01, Mann-Whitney *U* test); OM on the other hand had no effect (Fig. 5, *C* and *F*, *n* = 3). However, application of IAA and BrPy before Ca^2+^ influx resulted in inhibition of Ca^2+^ entry and dramatically reduced the plateau from which [Ca^2+^]*_i_* clearance was initiated and subsequently measured (Fig. 5, *B* and *D*). As a result, [Ca^2+^]*_i_* clearance could only be measured over a very narrow range of Ca^2+^ concentrations. Furthermore, the slowed Ca^2+^ influx meant it took longer to achieve a sufficient increase in [Ca^2+^]*_i_* from which to measure [Ca^2+^]*_i_* clearance rate. Using the current protocol where metabolic inhibitors were applied before Ca^2+^influx, this resulted in prolonged exposure (>15 min) to the glycolytic inhibitors before initiation of [Ca^2+^]*_i_* clearance. These confounding factors cast doubt on the validity of the [Ca^2+^]*_i_* clearance data obtained from MIA PaCa-2 cells with the current experimental design.

We, therefore, modified our [Ca^2+^]*_i_* clearance protocol to isolate the effects of the drugs on PMCA activity and to control for the duration of drug exposure before initiating [Ca^2+^]*_i_* clearance. To achieve this, 1 mm La^3+^ was applied in the absence of Ca^2+^ and EGTA at the peak of [Ca^2+^]*_i_* influx. This inhibited Ca^2+^ influx and efflux and thereby effectively clamped [Ca^2+^]*_i_* within the cell. Test reagents were applied simultaneously with La^3+^ at the peak of [Ca^2+^]*_i_* influx rather than before [Ca^2+^]*_i_* influx. After 5 min of treatment with a metabolic inhibitor, La^3+^ was rapidly removed using 1 mm EGTA, which has a high affinity for La^3+^ (36). The addition of EGTA and removal of La^3+^ allows initiation of [Ca^2+^]*_i_* clearance, and thus clearance can be assessed as before, without the confounding factors of a reduced Ca^2+^ influx rate and a prolonged drug exposure time influencing [Ca^2+^]*_i_* clearance.

**FIGURE 6.**
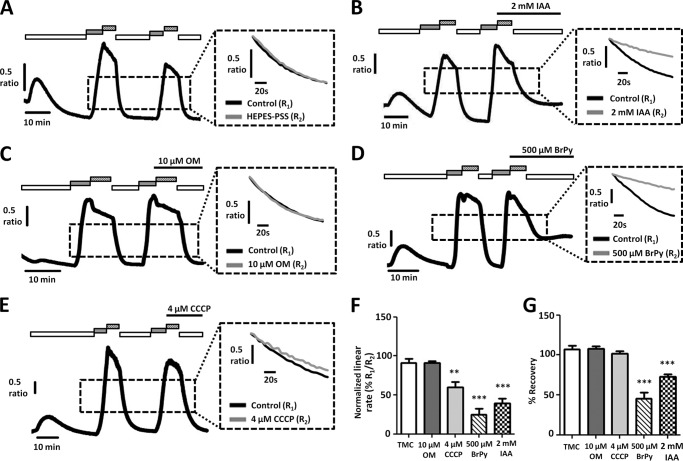
**Glycolytic inhibitors, but not mitochondrial inhibitors, inhibit PMCA activity in MIA PaCa-2 cells.** *A–E*, representative traces show a modified protocol *in situ* [Ca^2+^]*_i_* clearance assay (PMCA activity) in fura-2-loaded MIA PaCa-2 cells. Ca^2+^ influx was induced before application of test reagents to isolate their effects on clearance. CPA (30 μm) was applied in the absence of external Ca^2+^ with 1 mm EGTA (*white box*) or 20 mm Ca^2+^ (*gray box*) to induce store-operated Ca^2+^ influx. 1 mm La^3+^ was then applied at the peak of Ca^2+^ influx (*striped box*). Subsequent removal of external La^3+^ with 1 mm EGTA after 5 min allowed [Ca^2+^]*_i_* clearance. This influx-clearance phase was repeated, and metabolic inhibitors were applied during this second influx-clearance phase. Each *inset trace* shows expanded time courses comparing the second clearance phase (*gray trace*) with the first (*black trace*) in the presence of each metabolic inhibitor. *A*, TMC; *B*, 2 mm IAA; *C*, 10 μm OM; *D*, 500 μm BrPy; *E*, 4 μm CCCP. Linear clearance rate over 60 s during the second clearance phase was normalized to that of the first (% relative clearance). Recovery during the second clearance phase was normalized to the base-line [Ca^2+^]*_i_* before the first influx-clearance phase. *F*, normalized linear rate (± S.E.). *G*, recovery (± S.E.). **, *p* < 0.01; ***, *p* < 0.001 (Mann-Whitney *U* test), compared with TMC.

Similar to previous experiments, [Ca^2+^]*_i_* clearance rate during the second clearance phase of this amended protocol was reasonably well maintained at 91 ± 5% in control MIA PaCa-2 cells (Fig. 6, *A* and *F*, *n* = 9). Similar to PANC1 cells, BrPy (500 μm, Fig. 6*D*) and IAA (2 mm, Fig. 6*B*) both significantly decreased PMCA activity to 25 ± 8% (Fig. 6*F*, *n* = 6) and 40 ± 6% (Fig. 6*F*, *n* = 6), respectively, compared with corresponding time-matched control cells (*p* < 0.001 for both BrPy and IAA, Mann-Whitney *U* test). In contrast, 10 μm OM had no effect on [Ca^2+^]*_i_* clearance (*n* = 5, Fig. 6, *C* and *F*). Interestingly, however, CCCP caused a significant decrease in [Ca^2+^]*_i_* clearance (60 ± 7%, *p* < 0.001, *n* = 8, Mann-Whitney *U* test, Fig. 6, *E* and *F*).

We, therefore, also assessed the degree of recovery to base-line [Ca^2+^]*_i_* after each treatment. To measure recovery, the difference was calculated between the fura-2 ratio upon removal of La^3+^ during the second influx-clearance phase and the minimum value the steady state plateau subsequently reached. This was then normalized to the difference between the fura-2 ratio upon removal of La^3+^ during the second influx-clearance phase and the lowest fura-2 ratio value observed before the first influx-clearance phase. BrPy (500 μm, Fig. 6*D*) and IAA (2 mm, Fig. 6*B*) both significantly inhibited recovery to 45 ± 8% (*n* = 6) and 73 ± 3% (*n* = 6), respectively, compared with 107 ± 4% for the corresponding time-matched control cells (107 ± 4%, *n* = 9, *p* < 0.001, Mann-Whitney *U* test). In contrast, after treatment with the mitochondrial inhibitors, cells were able to fully recover [Ca^2+^]*_i_* to base-line values; neither OM (10 μm, Fig. 6*C*, 108 ± 3%, *n* = 5) nor CCCP (4 μm, Fig. 6*E*, 102 ± 4%, *n* = 7) had any effect on recovery compared with corresponding control cells. Taken together, these data suggest that glycolysis, but not mitochondrial metabolism, is critically important for both maintaining PMCA [Ca^2+^]*_i_* clearance rate and for ensuring full recovery to a low resting [Ca^2+^]*_i_*.

##### Inhibition of Glycolysis, but Not Mitochondrial Metabolism, Induces ATP Depletion in PDAC Cells

To assess the effects of glycolytic and mitochondrial inhibitors on ATP depletion, cells were treated with varying concentrations of BrPy, CCCP, IAA, or OM for 15 min before the addition of ViaLight® Plus ATP kit assay reagents (see “Experimental Procedures”). These concentrations were chosen because they were below, equal to, and higher than those used in the [Ca^2+^]*_i_* clearance experiments. Moreover, 15-min incubation was chosen because it was sufficient to induce [Ca^2+^]*_i_* overload and inhibition of the PMCA during our *in situ* [Ca^2+^]*_i_* clearance assays. Cells were also treated with a combination of all four metabolic inhibitors to induce maximum ATP depletion (ATP depletion mixture: BrPy, 500 μm; CCCP, 4 μm; IAA, 2 mm; OM, 10 μm). Raw luminescence counts obtained from those cells treated with the ATP depletion mixture were subtracted from each individual treatment before normalization to the luminescence counts of untreated control cells (%). On average, the raw luminescence count in control cells was 37,994 ± 5,976 in PANC-1 (*n* = 7) and 56,974 ± 10,865 in MIA PaCa-2 cells (*n* = 8) and in those treated with the ATP depletion mixture was 2,923 ± 519 in PANC-1 and 4,036 ± 1,032 in MIA PaCa-2 cells. Thus, the ATP depletion mixture reduced global ATP to 8 ± 1% in PANC-1 cells and 9 ± 3% in MIA PaCa-2 cells over 15 min. All statistical comparisons were made using a one-sample *t* test against the hypothetical control value of 100%.

**FIGURE 7.**
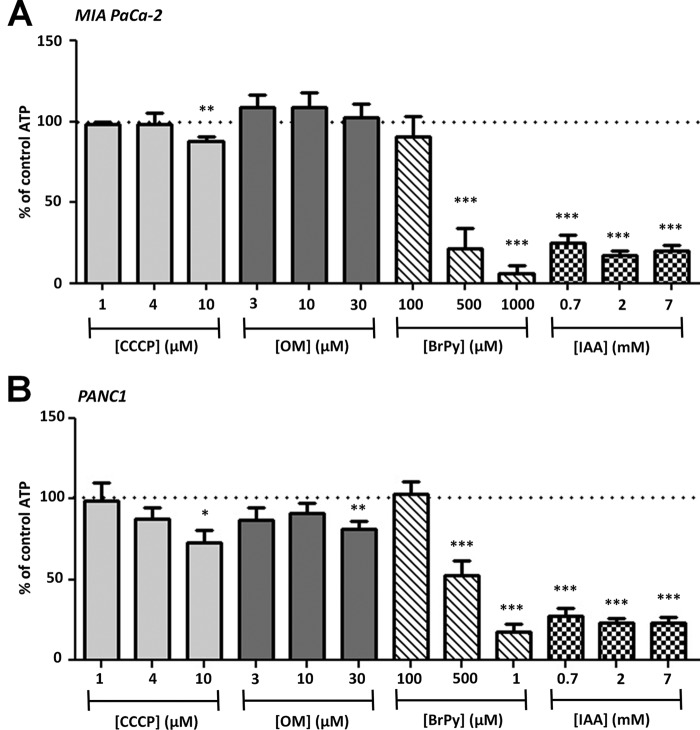
**Inhibition of glycolysis, but not mitochondrial metabolism, induces ATP depletion in PDAC cells.** MIA PaCa-2 (*A*) and PANC1 cells (*B*) were treated with either mitochondrial inhibitors (CCCP, 1–10 μm; OM, 3–30 μm) or glycolytic inhibitors (BrPy, 100–1000 μm; IAA, 0.7–7 mm) for 15 min. ATP depletion was determined using a luciferase-based luminescence assay. To determine ATP depletion (%), luminescence counts were normalized for each condition to untreated time-matched cells (total ATP). *n* = 8 for all experiments. *, *p* < 0.05; **, *p* < 0.01; ***, *p* < 0.001 (one-sample *t* test).

The glycolytic inhibitors BrPy and IAA caused a profound decrease in ATP in both MIA PaCa-2 and PANC1 cells. IAA caused a significant decrease in ATP at all three concentrations in both MIA PaCa-2 (0.7 mm, 25 ± 5%; 2 mm, 17 ± 3%; 7 mm, 20 ± 3%, *p* < 0.001, *n* = 8; Fig. 7*A*) and PANC1 cells (0.7 mm, 24 ± 3%; 2 mm, 23 ± 3%; 7 mm, 23 ± 3%, *p* < 0.001, *n* = 7; Fig. 7*B*). Likewise, the intermediate and high concentrations of BrPy caused a significant decrease in ATP in both MIA PaCa-2 (500 μm, 21 ± 13%; 1 mm, 6 ± 5%, *p* < 0.001, *n* = 8; Fig. 7*A*) and PANC1 cells (500 μm, 55 ± 10%; 1 mm, 19 ± 5%, *p* < 0.001, *n* = 7; Fig. 7*B*). Conversely, OM and CCCP had only a modest effect at concentrations higher than those used in our [Ca^2+^]*_i_* clearance experiments; 10 μm CCCP caused a significant decrease in ATP in both MIA PaCa-2 (89 ± 3%, *n* = 8, *p* < 0.01; Fig. 7*A*) and PANC1 cells (73 ± 8%, *n* = 8, *p* < 0.05; Fig. 7*B*), whereas 30 μm OM only caused a significant decrease in ATP in PANC1 cells (82 ± 5%, *n* = 8, *p* < 0.01; Fig. 7*B*).

These data suggest that glycolysis is the major mechanism of ATP synthesis in PANC1 and MIA PaCa-2 cells under these conditions and that inhibition of glycolysis is an effective means of inducing ATP depletion. On the other hand, it appears that mitochondrial respiration contributes far less to ATP production. Thus, the [Ca^2+^]*_i_* overload and PMCA inhibition observed after treatment with mechanistically distinct glycolytic inhibitors is most likely due to ATP depletion, as all these phenomena occur over a similar time course.

## DISCUSSION

The present study is the first to show that in human PDAC cell lines (PANC1 and MIA PaCa-2) both [Ca^2+^]*_i_* clearance and the maintenance of a low resting [Ca^2+^]*_i_* are critically dependent on glycolytic rather than mitochondrial metabolism. Inhibition of glycolysis inhibited PMCA activity and induced both substantial ATP depletion and an irreversible increase in cytosolic Ca^2+^ (“[Ca^2+^]*_i_* overload”) within minutes and ultimately caused cell death within 3–6 h. Inhibition of mitochondrial metabolism, on the other hand, had almost no effect on resting [Ca^2+^]*_i_*, PMCA activity, ATP depletion, or cell death. This is at variance with studies in non-cancerous pancreatic duct cells and the related pancreatic acinar cells, in which separate inhibition of either glycolysis or mitochondrial metabolism induced ATP depletion (37, 38) and inhibited PMCA activity (18). The present study also shows that the PMCA is the major Ca^2+^ efflux pathway in PANC1 and MIA PaCa-2 cells. Taken together, these data suggest that inhibition of glycolysis compromises PMCA activity as a result of ATP depletion, resulting in an irreversible [Ca^2+^]*_i_* overload and cell death. These findings are of potential therapeutic importance, as an increased dependence of the PMCA on glycolytically derived ATP may be a unique feature in tumor cells. PMCA activity is critical for the maintenance of a low resting [Ca^2+^]*_i_* (11, 39), and even if other [Ca^2+^]*_i_* clearance pathways such as SERCA are inhibited (for example, after treatment with CPA), [Ca^2+^]*_i_* will recover so long as the PMCA remains active. On the other hand, an inability to extrude [Ca^2+^]*_i_* via the PMCA would render [Ca^2+^]*_i_* overload an inevitability, as the endoplasmic reticulum capacity is finite. This leaves the PMCA as the “final gatekeeper” for the control of low resting [Ca^2+^]*_i_* in PDAC. Given that non-cancerous cells have a greater reliance on mitochondrial ATP production, targeting the glycolytic regulation of the PMCA may be an effective strategy to selectively kill PDAC cells while leaving non-cancerous cells unharmed. Furthermore, this may be relevant to other similar cancers where the PMCA is the major Ca^2+^ efflux pathway.

It has previously been described that PDAC cells exhibit the highly glycolytic phenotype characteristic of the Warburg effect (5, 40). Indeed, in the present study the glycolytic inhibitors IAA and BrPy both induced profound ATP depletion within 15 min, whereas the mitochondrial inhibitors OM and CCCP had no effect. In line with the present study, numerous studies have shown that BrPy causes ATP depletion in cancer cells (41). Although BrPy is reported to induce mitochondrial depolarization and thus impair mitochondrial function (42), this is unlikely to be the major mechanism by which BrPy induces ATP depletion in the present study, as CCCP also depolarizes ΔΨm (14) and had minimal effect on ATP depletion. This indicates that the mitochondrial ATP production capacity is of minor importance in these cells for maintaining ATP and suggests that BrPy depletes ATP by inhibiting glycolysis rather than impairing mitochondrial function. Furthermore, the mechanistically distinct glycolytic inhibitor IAA does not target the mitochondria yet depleted ATP regardless. We can, therefore, conclude from the degree of ATP depletion achieved by IAA and BrPy that glycolysis is the major source of ATP in both PANC1 and MIA PaCa-2 cells.

ATP depletion is well known to drive necrosis (43), and inhibition of glycolysis with BrPy would, therefore, be expected to induce cell death in glycolytically dependent PDAC cells as a result of ATP depletion. Indeed, in the present study, BrPy induced cell death in human PDAC cells, whereas inhibition of mitochondrial metabolism had no effect. Consistent with this, studies have shown that BrPy depletes ATP and induces cell death in highly glycolytic hepatocellular carcinoma cells without affecting normal hepatocytes (41). It has, however, been suggested that BrPy can exert its cytotoxic effects by inducing the release of reactive oxygen species (44) or cell death signaling factors (45) from the mitochondria. One counterargument to this, however, is that reactive oxygen species and cell death factors released from the mitochondria will most likely induce apoptosis rather than necrosis unless there is accompanying ATP depletion (14, 46). Apoptosis is an ATP-dependent process, and ATP depletion has been critically implicated in promoting necrotic cell death (47, 48). Moreover, in the present study, significant cell death was observed using propidium iodide, which is impermeant to apoptotic cells with an intact membrane. Similarly, BrPy induced cytotoxicity in hepatoma cells has been shown to be ATP-depletion-dependent (44). Although we cannot rule out these alternative mechanisms, we propose that the cytotoxic effects of BrPy in PDAC are driven primarily by ATP depletion.

In addition to ATP depletion, it is likely that the observed cell death was in part driven by [Ca^2+^]*_i_* overload. A sustained increase in [Ca^2+^]*_i_* is catastrophic for cells as it leads to the inappropriate activation of cytosolic enzymes such as proteases, phospholipases, and nucleases; it has long been known that a prolonged elevation in [Ca^2+^]*_i_* has cytotoxic and pathological effects (12, 14, 17). In the present study both PANC1 and MIA PaCa-2 cells were able to maintain a low resting [Ca^2+^]*_i_* after treatment with CCCP or OM, whereas treatment with either BrPy or IAA induced [Ca^2+^]*_i_* overload. Furthermore, [Ca^2+^]*_i_* overload was irreversible and associated with impaired ATP-induced [Ca^2+^]*_i_* responses, suggesting that glycolytic ATP is required for Ca^2+^ homeostasis and signaling. It is likely that profound ATP depletion would inhibit Ca^2+^ extrusion via the PMCA, thereby inducing the irreversible [Ca^2+^]*_i_* overload observed. However, because both ATP depletion and [Ca^2+^]*_i_* overload occurred over a similar timeframe (minutes), we are presented with a conundrum with respect to the sequence of events, as either could be expected to promote cell death. Furthermore, in addition to PMCA inhibition, ATP depletion would also be expected to inhibit the Na^+^/K^+^ ATPase. The Na^+^/K^+^ ATPase consumes a large fraction (>20%) of intracellular ATP to maintain the resting membrane potential of the cell (49), and thus its failure after ATP depletion would be expected to lead to cell membrane depolarization. This would result in the disruption of the maintenance of ion gradients, intracellular pH, and cell volume and decrease the driving force for Ca^2+^ entry. Because these effects could all contribute to the observed cell death, we cannot rule out inhibition of the Na^+^/K^+^ ATPase as a contributing factor to necrosis.

In addition to PMCA inhibition, it is likely that ATP depletion would also inhibit SERCA, leading to net endoplasmic reticulum Ca^2+^ leak, endoplasmic reticulum Ca^2+^ store depletion, and subsequent activation of store-operated Ca^2+^ entry. Thus, the effects of ATP depletion on SERCA and store-operated Ca^2+^ entry likely contributed to the rise in [Ca^2+^]*_i_* observed in our [Ca^2+^]*_i_* overload assays. However, inhibition of SERCA is unlikely to be the sole mechanism responsible due to the irreversible nature of the [Ca^2+^]*_i_* overload response. This is because inhibition of SERCA alone using CPA nearly always results in a transient rather than irreversible increase in [Ca^2+^]*_i_*. This strongly implies that [Ca^2+^]*_i_* efflux via the PMCA is also abolished during the irreversible [Ca^2+^]*_i_* overload response. Furthermore, although studies have shown that NCX is expressed in several human PDAC cell lines (CFPAC-1, PANC1, and Capan-1; Ref. 32), the present study shows that NCX has no role during our *in situ* [Ca^2+^]*_i_* clearance assay and that Ca^2+^efflux is achieved solely by the PMCA in PANC1 and MIA PaCa-2 cells. This makes glycolytic regulation of the PMCA all the more relevant to the survival phenotype of these cells, as PMCA inhibition would be expected to induce an irreversible [Ca^2+^]*_i_* overload.

The present study is also the first to show that glycolytically derived ATP is critical for PMCA function in PDAC cells and is, therefore, crucial for the maintenance of a low resting [Ca^2+^]*_i_* and thus cell survival. Interestingly, although OM had no effect on the rate or degree of [Ca^2+^]*_i_* clearance, CCCP caused inhibition of [Ca^2+^]*_i_* clearance in MIA PaCa-2 cells. However, despite this decrease in [Ca^2+^]*_i_* clearance rate, cells could still recover [Ca^2+^]*_i_* to base line. One explanation for this is that CCCP collapses ΔΨm, thereby decreasing the driving force for mitochondrial Ca^2+^uptake (50), which could appear as a modest reduction in [Ca^2+^]*_i_* clearance rate. Nevertheless, the PMCA was still capable of recovering a low resting [Ca^2+^]*_i_*, and CCCP had no effect on resting [Ca^2+^]*_i_*, ATP depletion, or cell death in our other experiments. In contrast to CCCP, [Ca^2+^]*_i_* never fully recovered to base line during our [Ca^2+^]*_i_* clearance assays on MIA PaCa-2 cells treated with BrPy or IAA.

Evidence suggests that glycolysis can contribute as much as half of the total ATP generated in tumor cells under aerobic conditions (9), and although the source of ATP to fuel the PMCA is likely unimportant provided the cytosolic ATP is maintained above a critical threshold, the glycolytic dependence of PDAC may render the PMCA exquisitely sensitive to inhibition of glycolysis in these cells. It is important to consider that the rate of ATP depletion after inhibition of glycolysis is likely to be much faster during our *in situ* [Ca^2+^]*_i_* clearance assay compared with our luciferase-based assays, as ATP is also being rapidly consumed due to the PMCA operating at full capacity. Nevertheless, inhibition of glycolysis resulted in an irreversible [Ca^2+^]*_i_* overload in resting PDAC cells, suggesting that glycolytic inhibition alone causes ATP depletion sufficient to compromise PMCA activity.

This poses the question as to what degree of ATP depletion would be required to inhibit the PMCA. Early studies in red blood cells showed that the PMCA has a high (μm) affinity for ATP (51). However, ATP regulation of the PMCA is more complex than previously thought (52) and can be influenced by [Ca^2+^]*_i_*, [Mg^2+^]*_i_*, calmodulin, and the phospholipid composition of the plasma membrane. For example, studies in human erythrocyte membranes have shown that the absence of phosphatidylserine decreases the affinity of the PMCA for ATP (53), presumably making the PMCA exquisitely sensitive to ATP depletion. Furthermore, functional studies in intact cells indicate that the PMCA is inhibited by the loss of phosphatidylserine from the inner leaflet of the plasma membrane (54), supporting evidence that phosphatidylserine plays a role in regulating the ATP sensitivity of the PMCA. It is important to note, however, that the majority of studies have used cell-free assays to examine the relationship between ATP and PMCA activity. It is, therefore, difficult to extrapolate the results of these studies to physiological PMCA activity in live intact cells, as dynamic changes in the lipid composition of the membrane, [Ca^2+^]*_i_*, and protein-protein interactions could profoundly alter these complex mechanisms. As such, the absolute threshold at which ATP depletion inhibits PMCA activity in intact cells is not currently known.

Despite this, evidence (including the current study) suggests that ATP depletion-induced inhibition of the PMCA impairs the maintenance of resting [Ca^2+^]*_i_*. We previously showed that an acute insulin-induced switch from mitochondrial to glycolytic metabolism in rat pancreatic acinar cells is sufficient to prevent ATP depletion and to protect PMCA activity in the face of oxidant-induced impaired mitochondrial function (18). This has important implications for the current study. Similarly, combined inhibition of both mitochondrial and glycolytic metabolism in mouse pancreatic acinar cells resulted in ATP depletion and corresponded with a decrease in Ca^2+^ extrusion rate (55). [Ca^2+^]*_i_* overload after inhibition of mitochondrial ATP production has also been shown to be prevented by maintaining cytosolic ATP via a patch pipette (13). Collectively these studies suggest that impairment of [Ca^2+^]*_i_* clearance due to ATP depletion can occur regardless of whether glycolysis or mitochondrial metabolism is perturbed, providing that global ATP depletion is sufficient. Importantly, however, in the present study, inhibition of glycolysis alone was sufficient to induce substantial ATP depletion in PDAC cells and resulted in both inhibition of the PMCA and a profound effect on resting [Ca^2+^]*_i_*. Unlike previous studies conducted in acutely isolated pancreatic cells, inhibition of mitochondrial metabolism had little or no effect on global ATP or [Ca^2+^]*_i_* clearance in the present study. Therefore, in addition to supporting the hypothesis that PMCA inhibition after metabolic stress is likely due to ATP depletion, the present study suggests that the PMCA in PDAC cells is sensitive to the depletion of glycolytic rather than mitochondria-derived ATP.

In light of these findings, it is tempting to speculate that glycolytic regulation of the PMCA may be an important pro-survival mechanism in PDAC tumors. Previous experiments using human erythrocyte membranes suggest that glycolytic enzymes associate with the plasma membrane (15) and that this affects their catalytic activity (56). Importantly, evidence suggests that these enzymes may be colocalized to the PMCA (15, 16), thereby potentially providing the PMCA with a privileged glycolytic ATP supply. Moreover, the aberrant metabolic profile and in particular the unique overexpression of key glycolytic enzymes in PDAC (5) may provide an “Achilles heel” that could be targeted to disrupt [Ca^2+^]*_i_* homeostasis in PDAC selectively.

Collectively the present study suggests that glycolytic ATP synthesis is critically important for maintaining PMCA activity and a low resting [Ca^2+^]*_i_* in human PDAC cell lines. Furthermore, these findings are translational and provide insights into a potentially new therapeutic avenue for the treatment of PDAC. Although targeting the glycolytic regulation of the PMCA in PDAC alone may be insufficient to eradicate the cancer unless part of a combination chemotherapy regimen, the link between the Warburg effect and [Ca^2+^]*_i_* homeostasis is an avenue previously unexplored. Moreover, glycolytic regulation of the PMCA might represent a critical pro-survival phenotype in other cancer types, particularly those derived from cells that rely on the PMCA as the major Ca^2+^ efflux pathway. Targeting key glycolytic enzymes that are up-regulated or uniquely expressed in highly glycolytic tumors such as PDAC could result in selective PMCA inhibition, thereby inducing [Ca^2+^]*_i_* overload-induced cell death in the tumor while sparing adjacent non-cancerous cells.

## Footnotes

3 The abbreviations used are:

PDAC: pancreatic ductal adenocarcinoma
PMCA: plasma membrane Ca^2+^ ATPase
[Ca^2+^]*_i_*: cytosolic Ca^2+^
NCX: Na^+^/Ca^2+^ exchanger
HEPES-PSS: HEPES-buffered physiological saline solution
NMDG: *N*-methyl d-glucamine HEPES-PSS
BrPy: 3-bromopyruvate
IAA: sodium iodoacetate
OM: oligomycin
CPA: cyclopiazonic acid
CCCP: carbonyl cyanide *m*-chlorophenyl hydrazine
AUC: area under the curve
TMC: time-matched control
PI: propidium iodide
SERCA: sarco(endo)plasmic reticulum calcium ATPase.
